# 18S is an appropriate housekeeping gene for *in vitro* hypoxia experiments

**DOI:** 10.1038/sj.bjc.6605754

**Published:** 2010-07-20

**Authors:** A Nagelkerke, H Mujcic, B Wouters, P N Span

**Affiliations:** 1 Department of Radiation Oncology, Radboud University Nijmegen Medical Centre, Nijmegen, The Netherlands; 2 Department of Laboratory Medicine, Radboud University Nijmegen Medical Centre, Nijmegen, The Netherlands; 3 Department of Radiation Oncology, University of Toronto, Toronto, Ontario, Canada

**Sir,**

We have read with great interest the study by Caradec *et al* (2010) in *British Journal of Cancer* into the variability in housekeeping gene expression under different oxygen tensions.

We concur with the authors that the choice of normalising gene can have important consequences on the results obtained, and should therefore be validated for the experimental setup, tissue, or cell line under investigation. Earlier, we have carried out a similar investigation of 13 different housekeeping genes in 80 different tumour and normal tissues, trying to compensate for both differences in tumour grade and RNA quality (de Kok *et al*, 2005). In the study of Caradec *et al*, however, the housekeeper is not used for compensation in differences in RNA quality, as this is much less of an issue in cell lines than in retrospectively analysed stored tumour tissue. Thus, the housekeeping gene suitable for normalisation of cell-line expression levels under different oxygen tensions should be primarily constant between different levels of hypoxia. For this reason, we have selected 18S to compensate for input, RT, and PCR efficiency for *in vitro* hypoxia experiments (Mujcic *et al*, 2009). Although this rRNA species is unsuitable for compensating for RNA degradation, as we have shown earlier (de Kok *et al*, 2005), and for normalising among cell lines, we have found it to be relatively stable at different oxygen tensions over short periods of time.

We would be interested in learning how 18S expression levels compared to the expression levels of the other genes tested by Caradec *et al.*
